# Muscle Synergy Analysis as a Tool for Assessing the Effectiveness of Gait Rehabilitation Therapies: A Methodological Review and Perspective

**DOI:** 10.3390/bioengineering11080793

**Published:** 2024-08-05

**Authors:** Daniele Borzelli, Cristiano De Marchis, Angelica Quercia, Paolo De Pasquale, Antonino Casile, Angelo Quartarone, Rocco Salvatore Calabrò, Andrea d’Avella

**Affiliations:** 1 Department of Biomedical and Dental Sciences and Morphofunctional Imaging, University of Messina, 98125 Messina, Italy; angelica.quercia@unime.it (A.Q.); acasile@unime.it (A.C.); 2 Laboratory of Neuromotor Physiology, IRCCS Fondazione Santa Lucia, 00179 Rome, Italy; a.davella@hsantalucia.it; 3 Engineering Department, University of Messina, Messina 98166, Italy; cristiano.demarchis@unime.it; 4 IRCCS Centro Neurolesi “Bonino Pulejo”, 98124 Messina, Italy; paolo.depasquale@irccsme.it (P.D.P.); angelo.quartarone@irccsme.it (A.Q.); roccos.calabro@irccsme.it (R.S.C.); 5 Department of Biology, University of Rome Tor Vergata, 00133 Rome, Italy

**Keywords:** motor coordination, musculo-skeletal redundancy, motor modules, neural disease, stroke, Parkinson’s disease, cerebral palsy, multiple sclerosis, myelopathy, brain tumor

## Abstract

According to the modular hypothesis for the control of movement, muscles are recruited in synergies, which capture muscle coordination in space, time, or both. In the last two decades, muscle synergy analysis has become a well-established framework in the motor control field and for the characterization of motor impairments in neurological patients. Altered modular control during a locomotion task has been often proposed as a potential quantitative metric for characterizing pathological conditions. Therefore, the purpose of this systematic review is to analyze the recent literature that used a muscle synergy analysis of neurological patients’ locomotion as an indicator of motor rehabilitation therapy effectiveness, encompassing the key methodological elements to date. Searches for the relevant literature were made in Web of Science, PubMed, and Scopus. Most of the 15 full-text articles which were retrieved and included in this review identified an effect of the rehabilitation intervention on muscle synergies. However, the used experimental and methodological approaches varied across studies. Despite the scarcity of studies that investigated the effect of rehabilitation on muscle synergies, this review supports the utility of muscle synergies as a marker of the effectiveness of rehabilitative therapy and highlights the challenges and open issues that future works need to address to introduce the muscle synergies in the clinical practice and decisional process.

## 1. Introduction

Gait is a complex task that requires the coordination of multiple muscles, driven by different cortical and spinal structures [1,2,3,4]. Neurological diseases can lead to impairments in the neuromuscular control of gait, with an increased risk of accidental falls [5] and the consequent reduced independence in performing activities of daily living, due to alterations in coordination strategies and in spatiotemporal gait parameters [6]. In this scenario, gait rehabilitation plays a crucial role, and recovering a functional gait is a fundamental step for independent living. Gait rehabilitation may require the action of physiotherapists alone, or it could additionally involve the use of rehabilitation devices. Gait assistive equipment comes in a variety of forms that can be roughly classified into two major categories that can be identified as follows [7,8]: alternative devices, which are used by patients with no movements and do not involve exercises for injured extremities, and augmentative devices, employed by people with limited mobility to generate movements or workouts with a rehabilitative purpose. Augmentative gait devices can be used on a treadmill, on foot-plates, overground through the use of mobile robotic bases [9], or they can be stationary [10]. The quantitative assessment of gait is of great importance to characterize the stage of a neurological pathology [11,12,13,14], to validate the beneficial effects of a therapy [15,16,17,18], or for the early detection of conditions such as risk of fall [19], dementia [20], and Parkinson’s disease [11]. The quantification of the motion of body segments in two or three dimensions is frequently exploited to estimate the spatiotemporal and kinematic parameters of gait [21]. This is usually carried out through marker-based [22] or marker-less [23,24] motion capture systems, even though the use of Inertial Measurement Units (IMUs) have started to gain popularity [25,26,27,28]. This kind of analysis is often complemented by the measurement of contact forces during the stance phase of gait to quantify impact forces, loading rates, propulsive and breaking forces, and to track variations in the center of pressure [29,30].

However, the main window on the physiological mechanisms underlying the neural control of gait is provided by electromyography (EMG), which allows to gain quantitative information regarding muscle coordination. Earlier EMG systems used cables to transmit recorded signals. This additional wiring represented a major limitation as it potentially reduced the subject’s range of motion. However, over the last two decades, EMG systems have been improved by the incorporation of technology that enables the data to be delivered wirelessly or kept in a data logger worn by the subject to detect relevant pathological features [31,32,33,34] or to drive robotic devices [35,36,37,38,39,40,41,42,43].

Concurrently, data analysis techniques have been proposed that allow us to investigate multi-muscle activity in order to quantitatively characterize the coordination among muscles acting across different joints. Muscle synergy analysis, i.e., the analysis of low-dimensional structures through the factorization of multi-muscle sEMG recordings, has gained popularity as it has been shown to provide relevant information regarding the neuro-mechanics of movement for a variety of motor tasks and motor impairment in neurological conditions [44,45,46,47,48,49,50,51,52,53,54,55,56,57]. Indeed, muscle synergies are suggested to derive from neural inputs that drive multiple muscles [58,59,60]. This idea is supported by the presence of synchronized oscillations within the firings of motor unit pools across different muscles, potentially stemming from cortical oscillations [61,62,63]. The sEMG signal represents the sum of action potentials induced in multiple muscle fibers [64,65]. Motor unit action potentials have been modeled as electromagnetic resonant modes [66], and their spectral characteristics [67] are known to change during fatiguing tasks [36,68,69], with aging [70], and at different muscle temperatures [71]. The synchronous modulation of motor unit pools is thought to reduce kinematic noise [72] and the existence of a common drive has been suggested to respect both the size principle [73,74,75,76] and the onion skin principle [77].

Many previous studies have shown that muscle coordination during human gait can be well described by the combination of a small number of muscle synergies and that each synergy is associated with specific biomechanical functions of gait in various conditions [53,78,79,80,81,82]. There is relatively vast literature analyzing muscle synergies in gait in physiological and pathological conditions, indicating how changes in the muscle synergy dimensionality and spatiotemporal structures can account for several behavioral correlates and clinical scales as well. This body of literature collectively suggests that muscle synergy analysis might be a good quantitative tool to investigate the neural correlates of gait performance and functional gait recovery.

Following the acknowledgment of motor synergies as a potential tool to compactly index motor functions [83,84,85], reviews have been published that examine the alteration of synergies after neurological diseases such as Parkinson’s disease [86], stroke [87,88], or neurodevelopmental disorders [89]. However, to the best of our knowledge, no review to date has comprehensively examined the alteration of muscle synergies identified during locomotion tasks after rehabilitation therapy. If muscle synergies capture the neurophysiological mechanisms underlying the control of gait and provide a reliable indicator of gait performance, they should also highlight specific changes in motor coordination during motor recovery. Therefore, this work aims to systematically review the literature to assess the state of the art in research on the use of muscle synergies during a locomotion task, as a quantitative tool to analyze the efficacy of rehabilitation therapy.

## 2. Materials and Methods

### 2.1. Search Strategy

A systematic search strategy was conducted using the electronic databases of PubMed, Scopus, and Web of Science and performed on the 1 April 2024. The time of publication was restricted to the interval between January 2011 and March 2024. The lower bound of January 2011 was that of the publication date of the first study that suggested muscle synergies as a tool for clinicians to assess healthy and pathological muscle activity [83]. As shown in Table 1, the searches involved a combination of the following words only in the title or abstract: “Gait”, or any word beginning with “walk” or “locomot”; “Therapy”, or “training”, or any word beginning with “rehabilit” or “neurorehabilit”; “synergy”, or “synergies”, or “muscle coordination”, or “motor module”, or “motor modules”, or “primitive”, or “primitives”.

### 2.2. Study Eligibility: Inclusion and Exclusion Criteria

Studies were retained if the following eligibility criteria were met: (1) a pathological condition was investigated; (2) a therapy, which was finalized to recover gait motor function, was administered; (3) the muscle synergies of the patients were assessed before and after the therapy; (4) studies followed clear and reproducible methodological stages [90], and studies were excluded when the therapy was not adequately specified, the task was not clear and reproducible, or the assessment of muscle synergies did not include the lower limb; (5) the study was published in English in peer-reviewed journals. Reviews, meta-analysis articles, perspective/position papers, editorials, commentaries, and conference papers were excluded, but case reports were retained.

### 2.3. Study Selection and Data Extraction

**Figure 1:**
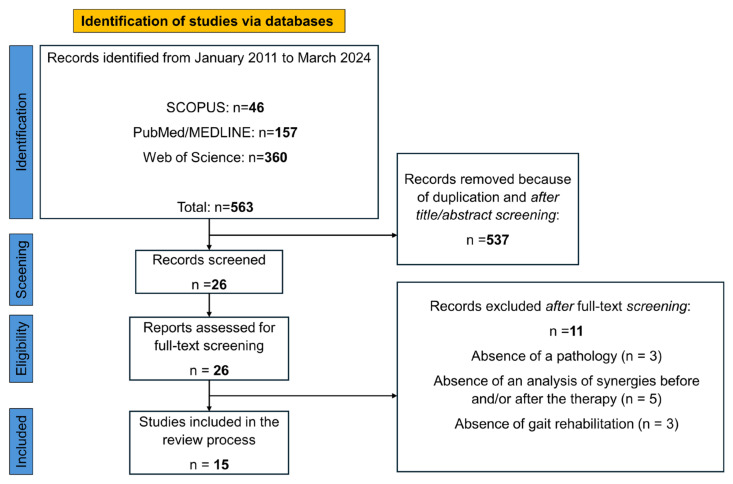
PRISMA flowchart for study inclusion/exclusion.

In compliance with the PRISMA statement [91,92], the eligibility of potentially relevant studies was based on title and abstract adherence to inclusion/exclusion criteria, and the screening was conducted by three authors independently (D.B., C.D., and Angelica Quercia). Full texts were then retrieved and evaluated thoroughly to confirm eligibility based on the described inclusion and exclusion criteria. Conflicts were resolved by discussions among the authors. Reference lists of the included studies were manually screened to identify additional relevant studies. From the retained studies, we extracted the following information and imported them into an Excel spreadsheet: First author, year of publication, the number of patients, the assessed pathology, the collected muscle set (for details, see Table 2), the type of task during which muscle synergies were extracted, the type of training and its duration, the algorithm used to extract the muscle synergies and define their number, the metrics adopted to compare synergies extracted before and after the training were tabulated for each study. The number of patients and the number of control participants enrolled in each study were both recorded in the spreadsheet. Additionally, a check on the inclusion of healthy control participants, the acquired clinical scales, and the presence of other kinematic measures was conducted. Figure 1 shows the PRISMA flowchart for study inclusion/exclusion.

## 3. Results

### 3.1. Selected Studies

Our search query returned a total of 563 studies, of which 26 full-text articles were retained and further screened based on their title and abstract and the inclusion/exclusion criteria. Manual reference list screening did not result in any additional studies. A total of 15 articles passed this further screening and were thus included in this review.

### 3.2. Study Characteristics

#### 3.2.1. Patients

Most of the retained studies (9 out of 15) investigated the effect of physical gait rehabilitation on the muscle synergies of stroke survivors [93,94,95,96,97,98,99,100,101]. The other studies investigated Parkinson’s disease [102,103], cerebral palsy [104], multiple sclerosis [105], myelopathy [106], and brain tumor [107].

Four studies enrolled a large population of patients (≥20 patients) [96,99,100,103], while two studies were case reports [106,107], and two other studies only enrolled two patients each [94,95].

Three studies included as control groups patients who underwent conventional therapy [99], cognitive training [100], or treadmill training with body weight support [97].

Seven studies also enrolled healthy control participants to define a set of representative healthy muscle synergies sets and activation profiles for reference data [94,95,96,97,101,103,105], five of which selected age-matched healthy controls [95,96,97,103,105].

#### 3.2.2. Task

During walking, the changes in the muscle synergies were investigated overground in 10 studies [94,95,97,98,99,100,102,103,105,106] but also on a treadmill [96,104,107]. Both overground and treadmill conditions were investigated in one study [101]. In addition to gait, in two studies, muscle synergies were also explored during a reactive balance task [102] and a recumbent cycling task [93].

#### 3.2.3. Training and Clinical Evaluation

In the 15 retained studies, patients underwent rehabilitative training for an average of 4 weeks (range: 3–12 weeks). Rehabilitative sessions (14 on average; range: 9–36) were administered 2 to 5 times a week, and each session lasted 33 min on average (range: 5–90 min).

In 6 out of our 15 selected studies [97,98,99,101,105,107], gait rehabilitative training of the lower limbs of patients was based on robotic exoskeletons [108,109]. Lower-limb exoskeleton-assisted training was mainly used for post-stroke patients [97,98,99,101], in children with cerebral palsy [104] and in cases of thoracic myelopathy [106]. In 4 of the selected studies, multichannel Functional Electrical Stimulation (FES) was applied as a rehabilitation method combined with walking [94,95,107] and cycling [93]. FES is considered an effective intervention for lower-limb rehabilitation, particularly suitable in stroke patients, as the combination of FES with walking or cycling can enhance motor learning and plasticity, improve locomotion ability, and strengthen lower limb muscles and motor coordination [110,111]. In the absence of severe musculoskeletal and neurologic pathologies that could influence gait, treadmill walking sessions can also be used for locomotor rehabilitation [112,113]. In the retained studies, treadmill training sessions were administered both in stroke patients [96] and patients with multiple sclerosis [105]. Finally, in three out of the 15 studies [101,103,104], the rehabilitation protocol did not involve an assistive device. One study [100] investigated muscle activation patterns after trunk training in stroke patients to provide new insights in gait recovery. Another study [102], instead, investigated changes in the neuromuscular control of gait and balance after dance-based rehabilitation in Parkinson’s patients, specifically, an Adapted Tango (AT) dance. Finally, a third study [103] investigated the alteration in muscle synergies after a bilateral deep brain stimulation of the subthalamic nucleus.

Across the retained studies, participants were evaluated at baseline (T0) and after an intervention period (T1) with the following validated clinical scales used to measure patient motor functions: Functional Independence Measure-Motor General (FIM-M) [114]; Functional Independence Measure-Locomotion (FIM-Locomotion) [114]; Functional Ambulation Categories (FACs) [115]; The Motricity Index (MI) [116]; Fugl-Meyer Assessment, Lower Extremity (FMA-LE) [117]; Barthel Index (BI) [118]; Functional gait assessment (FGA) [119]; the Berg Balance scale (BBS) for standing balance [120]; Mini Best test (MBT), which evaluated the dynamic balance [121]; Fullerton Advanced Balance scale (FAB) [122]; Dynamic Gait Index (DGI) [123]; the 2 Minute Walking Test (2MWT) for gait endurance [124]; the 10 m timed walk (10MTW) for gait speed [125]; time up and go (TUG) [126]; the 6 min walk test (6MWT) [124]; Tinetti Performance Oriented Mobility Assessment (POMA) [127]; the Brunnstrom recovery stage (BRS), which evaluated impairment of the lower limb [128]; Trunk Control Test (TCT) [129]; and Trunk Impairment Scale (TIS) [130]. The motor function of children with cerebral palsy in [104] was evaluated following the Gross Motor Function Classification System (GMFCS) [131] to establish the ability to walk and which lower limb was most affected.

#### 3.2.4. Muscles

Across the selected studies, the EMG activity was collected either unilaterally or bilaterally. Given a set of available EMG sensors, a unilateral arrangement allows to collect the activity of more muscles in a single leg, and therefore, it provides a finer characterization of the locomotor patterns which may allow for a better assessment of intra-subject variability [82]. On the contrary, a bilateral sensor arrangement allows to investigate gait asymmetries occurring as a consequence of several diseases [132]. Six out of the 15 identified studies collected muscle activity unilaterally [94,95,97,102,104,105], and seven studies collected muscle activity bilaterally [93,96,98,99,101,106,107]. One study [100] collected unilaterally the activity of six leg muscles and bilaterally the activity of one trunk muscle (i.e., the erector spinae), while another study [103] collected unilaterally the activity of eleven leg muscles and bilaterally the activity of one trunk muscle (i.e., the longissimus dorsi).

**Figure 2:**
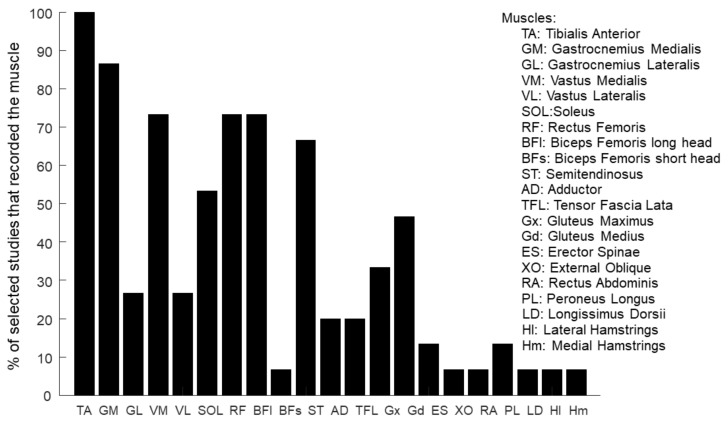
Percentage of the selected studies in which the activity of a muscle is collected.

Muscle selection varied across studies. In Table 2, the number and the identity of the muscles included in each study are reported, while in Figure 2, the percentage of studies which included each muscle is reported. Due to the redundancy of the musculoskeletal system [133] or its abundance [134], different studies selected different muscles with similar function, e.g., while nine studies collected the activity only from Vastus Medialis muscle (VM), three studies collected the activity from the Vastus Lateralis muscle (VL) whose action is the same as the VM. Moreover, one study [103] collected the activity from the lateral and medial hamstring without specifying whether the activity was from the semitendinosus or the semimembranosus muscles (medial hamstring) or the short or long head of the biceps femoris (lateral hamstring).

**Table 2:** Muscles that were collected from the selected studies. An ‘X’ in correspondence to the column related to a muscle indicates that the muscle was acquired. The muscles were Tibialis Anterior (TA), Gastrocnemius Medialis (GM), Gastrocnemius Lateralis (GL), Vastus Medialis (VM), Vastus Lateralis (VL), Soleus (SOL), Rectus Femoris (RF), Biceps Femoris long head (BFl), Biceps Femoris short head (BFs), Semitendinosus (ST), Adductor (AD), Tensor Fascia Lata (TFL), Gluteus Maximus (Gx), Gluteus Medius (Gd), Erector Spinae (ES), External Oblique (XO), Rectus Abdominis (RA), Peroneus Longus (PL), Longissimus Dorsii (LD), Lateral Hamstrings (Hl), and Medial Hamstrings (Hm). The ‘number of muscles’ column reports how many muscles were collected on a single side (unilaterally, U) and on both sides (bilaterally, B).

	Muscles																					
Reference	TA	GM	GL	VM	VL	SOL	RF	BFl	BFs	ST	AD	TFL	Gx	Gd	ES	XO	RA	PL	LD	Hl	Hm	Number
Allen et al., 2017 [102]	X	X	X	X		X	X	X				X		X	X	X	X	X				13 U
Ambrosini et al., 2020 [93]	X	X			X	X	X	X	X			X	X									9 B
Conner et al., 2021 [104]	X				X	X				X												4 U
Ferrante et al., 2016 [94]	X	X		X			X	X		X			X									8 U
Ghislieri et al., 2023 [103]	X		X	X		X	X			X		X		X				X	X	X	X	12U + 1B
Jonsdottir et al., 2020 [105]	X	X	X	X		X	X			X				X								8 U
Kadone et al., 2020 [106]	X	X		X						X			X									5 B
Kinugawa et al., 2022 [107]	X	X					X	X														4 B
Lim et al., 2021 [95]	X	X		X			X	X		X	X			X								8 U
Routson et al., 2013 [96]	X	X		X		X	X	X		X				X								8 B
Srivastava et al., 2016 [97]	X	X	X	X	X	X	X	X		X				X								10 U
Tan et al., 2020 [99]	X	X		X				X			X		X									6 B
Tan et al., 2018 [98]	X	X		X				X			X		X									6 B
Van Criekinge et al., 2021 [100]	X	X			X		X	X		X					X							6 U + 1 B
Zhu et al., 2021 [101]	X	X		X		X	X	X		X				X								8 B

#### 3.2.5. Muscle Synergies Extraction

In all the retained studies, muscle synergies were analyzed within the framework of the spatial (or synchronous) model, in which the muscle activity ***m*** of *M* muscles can be represented as the linear combination of a set of *N* < *M* time-invariant modules ***W*** modulated by time-varying activation profiles ***C***, as follows:

$$m\left(t\right)=\overset{N}{\underset{i=1}{\sum }}w_{i}\;c_{i}\left(t\right)+\varepsilon (t)$$

In which ***m***(*t*) is a vector of EMG data samples of all the recorded muscles at time *t*, ***w****_i_* is the *i*-th synergy vector, *c_i_*(*t*) is the synergy activation coefficient of the *i*-th synergy at time *t*, and ***ε***(*t*) is additive noise vector at time *t*. In all studies but one, muscle synergies were extracted through a Non-negative Matrix Factorization (NMF) algorithm [135], which decomposes the muscle activation data matrix $M=\left[m\left(t_{1}\right)\ldots m\left(t_{T}\right)\right]$, whose dimensions are [*M* × *T*] where *M* is the number of muscles and *T* the number of time samples, into two matrices such that M = ***WC***+ ***ε***, where ***W*** is an [*M* × *N*] matrix with *N* muscle synergies, and ***C*** is an [*N* × *T*] matrix of synergy activation coefficients. One study [93] implemented an algorithm, the Weighted Non-negative Matrix Factorization (WNMF) [136], that differs from the traditional NMF as it assigns each data sample a weight (1 =  EMG present, 0  =  EMG absent), to accommodate clinical data that contain poor or missing EMG channels.

The number of muscle synergies, which is a free parameter of the factorization algorithm, was defined in all studies but one, according to the uncentered Variance Accounted For (*VAF*), which measures the quality of the experimental EMG data with the extracted synergies:

$$VAF=100\cdot \left(1-\frac{\sum_{t}{\left(m_{t}-\left(Wc_{t}\right)\right)}^{2}}{\sum_{t}{\left(m_{t}\right)}^{2}}\right)$$

One study [103] used a centered *VAF*, otherwise called the coefficient of determination ($R^{2}$), defined as follows:

$$R^{2}=100\cdot \left(1-\frac{\sum_{t}{\left(m_{t}-\left(Wc_{t}\right)\right)}^{2}}{\sum_{t}{\left(m_{t}-\bar{m}\right)}^{2}}\right)$$

where $\bar{m}$ is the mean of the observed EMG data and retained the number of synergies at which the $R^{2}$ vs. number of synergies curve achieves the highest curvature [137].

Seven studies identified the number of synergies as the set whose *VAF* was higher than 90% [93,96,97,100,102,105,106]. Three studies [94,98,99] added to the condition of *VAF* > 90% a second condition, by which a new synergy, added to the identified set, did not increase the *VAF* more than 5%. One study [101] exploited a ‘three-way *VAF* > 90%’, which imposed that the overall *VAF*, calculated with all muscles through the entire gait cycle, each of the *VAF* values separately calculated for each muscle throughout the entire gait cycle, and each of the *VAF* values calculated with all muscles within six separate gait phases were higher than 90%. In contrast, two studies [104,107] calculated the total variance accounted for by one synergy from the EMG data (*VAF1*), and one study [95] reconstructed the EMG signals collected from patients with four synergies extracted from healthy participants.

#### 3.2.6. Muscle Synergy Analysis and Improvement-Related Metrics

To assess the effectiveness of the adopted rehabilitation therapy, the retained studies relied on a set of metrics related to the spatiotemporal structure of the extracted muscle synergies and compared their evolution along the rehabilitation process, mostly comparing the T0–T1 modifications in these metrics before (T0) and after (T1) the therapy and sometimes comparing them with the metrics of healthy participants, when available.

- Clinical evaluation

As shown in Table 3, overall, patients improved in all clinical scales after the rehabilitation protocols. The most sensitive scales to clinical improvement in motor function resulted in the Functional Independence Measure-Locomotion (FIM-Locomotion) [114], the 10 m timed walk (10MTW) for gait speed [125], and 6 min walk test (6MWT) [124]. However, only [93] investigated correlations between muscle synergies and clinical scales. The authors found from moderate to high correlations between BBS, TCT, and motor subscale of FIM and VAF1 of the affected and unaffected leg.

- Number of synergies

Among the analyzed parameters, the number of extracted synergies was the most commonly adopted metric (see Table 4), as 10 out of the 15 retained studies used the number of extracted synergies as a marker of the complexity of the modular organization [79] to assess rehabilitation effectiveness [93,94,96,98,99,100,101,102,103,105].

No accordance was identified among studies in terms of changes in the number of muscle synergies after the intervention. Some of the studies on stroke patients reported that an increase in the number of muscle synergies reflected an increase in performance [94,96,100] but other studies on stroke patients [93] and on patients with multiple sclerosis [105] or with Parkinson’s disease [103] did not show such change after the therapy. One study even reported a reduction in the number of muscle synergies for a subset of participants [102].

- Spatial and temporal organization

A total of 10 out of the 15 studies made quantitative assessments of the spatial composition of the extracted muscle synergies, either by comparing their spatial structure [93,94,95,96,97,98,99,100,101,103,105] or their degree of bilateral symmetry [98,99] to that of healthy individuals. The same studies also used the temporal activation coefficients to define quantitative metrics, either by calculating the temporal symmetry between sides or by measuring the similarity with the synergy activation pattern of healthy controls. A subset of studies (4 out of 15) defined VAF-based metrics to compactly indicate the complexity of muscle coordination with the VAF explained by one synergy [93,104,106,107] or two and three synergies [106]. One study constructed specific metrics on the synergy vectors, assessing generalizability, sparsity, and variability [102].

An increase in the complexity after rehabilitation was identified in patients with brain tumor [107] or cerebral palsy [104] but not in stroke patients [93] or patients with myelopathy [106]. Finally, an increase in the synergy coefficients within gait subphases was correlated with performance increase in patients with multiple sclerosis [105].

Some neurological diseases, such as unilateral stroke, led to a kinematical asymmetry in the lower limbs during locomotion [138], which is reflected in an asymmetry in muscle synergies [139]. Therefore, an improvement in the synergy symmetry, which was accompanied by an improvement in performance, was demonstrated by [98], or a synergy timing symmetry, not accompanied by improvements in clinical scores, was identified by [99].

## 4. Discussion

The goal of the present review was to assess the state of the art of the investigation of the effect of gait rehabilitation in patients with neurological diseases in terms of changes in the organization and recruitment of muscle synergies. Even though muscle synergies as a tool to investigate motor coordination was introduced over two decades ago [140,141,142] and despite several studies demonstrating that neurological patients showed altered muscle synergies with respect to healthy participants [51,86,143,144,145,146] and that synergies were proposed as a potential candidate marker for the quantitative assessment of neurological pathologies [143,147], only a few studies have specifically investigated the alteration of muscle synergies after rehabilitation. Specifically, we found only 15 studies in the last 13 years that investigated the effect of gait rehabilitation therapies on muscle synergies in neurological patients and to what extent the changes in muscle synergies are related to clinical improvements.

Fourteen out of the 15 selected studies reported a modification of the muscle synergies after gait rehabilitation, and only one study [106] identified no clear effect on muscle synergies. Most of the studies that involved a control group demonstrated that rehabilitation makes the muscle synergies more similar to those of healthy participants in terms of structure. In contrast, the temporal patterns of activation of the muscle synergies identified in patients with multiple sclerosis after rehabilitation differed from those of healthy controls when walking at comparable speeds [105]. An improvement in the synergy symmetry [98], or timing synergy symmetry [99], were also identified.

No accordance could be found across studies, in terms of changes in the number of muscle synergies. While some studies on stroke patients demonstrated an increase in the number of muscle synergies, also related to a performance increase [94,96,100], other studies that enrolled patients with multiple sclerosis [105], Parkinson’s disease [102], or stroke patients [93] either found no change or even a reduction in the number of muscle synergies [102]. Moreover, an increase in the number of synergies after rehabilitation was identified in patients with brain tumor [107] or cerebral palsy [104] but not in stroke patients [93] or patients with myelopathy [106]. Finally, an increase in the synergy coefficients within gait subphases was correlated with performance increase in patients with multiple sclerosis [105].

Overall, this review supports the hypothesis that modifications in the muscle synergies can index the progression of the rehabilitation process in an interpretable and quantitatively measurable manner.

Discrepancies were found among the studies. These discrepancies may be a consequence of the investigated neurological pathologies, which differ among the studies, and therefore may differently influence motor control and muscle synergies. Moreover, these discrepancies may reflect the lack of standardized protocols for processing and investigating muscle synergies and to compare them with those extracted from healthy participants.

When characterizing the modular control of locomotion in healthy subjects, four-to--five muscle synergies are typically extracted, and they are usually related to four specific biomechanical sub-functions along the gait cycle [79]:

- M1—a knee-hip extensor module, activated during early stance, serving as body support and weight acceptance
- M2—a calf plantar-flexor muscle module, activated during late stance, with forward propulsion, body support, and swing preparation function
- M3—an ankle dorsiflexion module, activated during early swing, contributing to the ground clearance of the foot
- M4—a knee flexor module, activated during late swing, to decelerate the leg an prepare heel strike

Despite the previously discussed large variability both in adopted methodologies and pathological conditions across studies, few invariances could be identified regarding the effect of rehabilitation therapy on muscle synergies. Indeed, regardless of the adopted metric to characterize the change, all those studies showing modifications in the spatial organization or temporal recruitment of muscle synergies after the therapy [70,71,72,73,77,78,82] reported a modification related to the plantarflexion module M2 and/or the knee flexor module M4, likely indicating an effect on forward propulsion and balance.

A recommendation for future research is to define standardized protocols and algorithms for the characterization of synergy structures and patterns in neurological patients, and for their comparison with healthy participants [148]. This would allow for a systematic characterization and comparison of the muscle synergies of patients with different pathologies and different levels of motor impairment.

Moreover, this review underlines the need for further research on muscle synergy analysis in the assessment of neurological patients’ rehabilitation, before it can be fully transferred to the clinical practice as a marker of the progression of the rehabilitation and as a support to clinical decision making [85], which would provide clinicians and therapists with a novel instrument to assess the efficacy of a therapy and whether and how it should be changed, not only by analyzing movement kinematics and kinetics but also through the lens of the underlying neural control strategies.

## 5. Conclusions

The present review strongly suggests that muscle synergy analysis has a very high potential as a tool to quantitatively assess the efficacy of rehabilitation therapies in neurological patients. However, it also highlights the significant lack of studies specifically investigating the effect of physical rehabilitation on muscle synergies. In the last 13 years, only 15 studies have examined the alteration of muscle synergies after physical rehabilitation during locomotion tasks. This indicates that the potential of muscle synergy analysis remains largely untapped as it has been scarcely used in clinical studies so far. For this promising approach to move to the clinical practice, the scientific community working on these topics should spend a greater effort in defining a methodological standardization of the assessment protocols and algorithms for the extraction and description of muscle synergies, together with the creation of an extensive publicly available database of the synergies identified in patients with different neurological pathologies and different levels of impairment. This would facilitate the standardization of the adopted procedures, together with the related metrics to quantify the effect of motor rehabilitation on the muscle synergies of a patient. This would provide clinicians and physiotherapists with a novel tool to be used as a marker of the effectiveness of therapy, as well as a source of information to develop innovative therapies.

## Figures and Tables

**Table 1:** Search query used in all systematic searches (PubMed version shown in table).

Search Query (PubMed, Scopus, WoS)	(“gait”[Title/Abstract] OR “walk*”[Title/Abstract]) AND (“therapy”[Title/Abstract] OR “rehabilit*”[Title/Abstract] OR “neurorehabilit*”[Title/Abstract] OR “training”[Title/Abstract]) AND (“muscle synerg*”[Title/Abstract] OR “synergies”[Title/Abstract] OR “muscle coordination”[Title/Abstract] OR “motor module*”[Title/Abstract] OR “primitive*”[Title/Abstract])

**Table 3:** Effect of the rehabilitation therapy on measured clinical scales.

	Clinical Scales		
	Improved	Not Improved	Not Altered
Allen et al., 2017 [102]	UPDRS-III, BBS, FAB, DGI, TUG, 6MWT		
Ambrosini et al., 2020 [93]	MI, TCT, BBS, FIMM		
Conner et al., 2021 [104]			
Ferrante et al., 2016 [94]	mini best test, Fugl-Meyer motor		
Ghislieri et al., 2023 [103]	UPDRS-III, FAB		MMSE
Jonsdottir et al., 2020 [105]	2MWT, 10MWT		BBS
Kadone et al., 2020 [106]	FIM motor, Barthel, FAC	10MWT	
Kinugawa et al., 2022 [107]	10MWT	FMA, BRS	
Lim et al., 2021 [95]	10MWT		BBS
Routson et al., 2013 [96]	FMA		DGI
Srivastava et al., 2016 [97]	FMA, FGA, 6MWT, TUG		
Tan et al., 2020 [99]	FIM motor, FIM locomotion, FMA lower ex		
Tan et al., 2018 [98]	FIM motor, FIM locomotion, FMA lower ex		
Van Criekinge et al., 2021 [100]	FAC, TIS, POMA Tinetti,		Barthel
Zhu et al., 2021 [101]	10MWT, 6MWT	TUG	

**Table 4:** Type of rehabilitation therapy (R = robotic based, F = FES-based, O = other techniques) and its effect of the rehabilitation therapy on muscle synergies characteristics (+: improvement post-rehabilitation, −: no improvement post-rehabilitation, n/a: comparison not performed).

	Type of Therapy	Number of Synergies	Spatial Synergies	Temporal Activations	Coordination Symmetry
Allen et al., 2017 [102]	O	−	+	n/a	n/a
Ambrosini et al., 2020 [93]	F	−	+	+	−
Conner et al., 2021 [104]	R	+	n/a	n/a	n/a
Ferrante et al., 2016 [94]	F	+	n/a	n/a	n/a
Ghislieri et al., 2023 [103]	O	+	+	+	n/a
Jonsdottir et al., 2020 [105]	O	−	−	+	n/a
Kadone et al., 2020 [106]	R	−	n/a	n/a	n/a
Kinugawa et al., 2022 [107]	F	+	n/a	n/a	n/a
Lim et al., 2021 [95]	F	n/a	+	+	n/a
Routson et al., 2013 [96]	O	+	+	+	n/a
Srivastava et al., 2016 [97]	R	n/a	+	−	n/a
Tan et al., 2020 [99]	R	+	n/a	n/a	+
Tan et al., 2018 [98]	R	−	n/a	n/a	+
Van Criekinge et al., 2021 [100]	O	−	+	n/a	n/a
Zhu et al., 2021 [101]	R	−	−	+	n/a

## Data Availability

Data can be made available on request by the authors.
